# Patient-Centered eHealth Interventions for Children, Adolescents, and Adults With Sickle Cell Disease: Systematic Review

**DOI:** 10.2196/10940

**Published:** 2018-07-19

**Authors:** Sherif M Badawy, Robert M Cronin, Jane Hankins, Lori Crosby, Michael DeBaun, Alexis A Thompson, Nirmish Shah

**Affiliations:** ^1^ Division of Hematology, Oncology and Stem Cell Transplant Ann & Robert H Lurie Children's Hospital of Chicago Chicago, IL United States; ^2^ Department of Pediatrics Feinberg School of Medicine Northwestern University Chicago, IL United States; ^3^ Department of Pediatrics, Division of Hematology and Oncology Faculty of Medicine Zagazig University Zagazig Egypt; ^4^ Department of Biomedical Informatics Vanderbilt University Medical Center Nashville, TN United States; ^5^ Department of Internal Medicine Vanderbilt University Medical Center Nashville, TN United States; ^6^ Department of Hematology St Jude Children’s Research Hospital Memphis, TN United States; ^7^ Cincinnati Children's Hospital Medical Center Cincinnati, OH United States; ^8^ Department of Psychology University of Cincinnati Cincinnati, OH United States; ^9^ Division of Hematology and Oncology, Department of Pediatrics Vanderbilt-Meharry Center for Excellence in Sickle Cell Disease Vanderbilt University Medical Center Nashville, TN United States; ^10^ Division of Hematology Duke University School of Medicine Durham, NC United States

**Keywords:** sickle cell, self-management, eHealth, mHealth, interventions, internet, anemia, sickle cell, telemedicine

## Abstract

**Background:**

Sickle cell disease is an inherited blood disorder that affects over 100,000 Americans. Sickle cell disease–related complications lead to significant morbidity and early death. Evidence supporting the feasibility, acceptability, and efficacy of self-management electronic health (eHealth) interventions in chronic diseases is growing; however, the evidence is unclear in sickle cell disease.

**Objective:**

We systematically evaluated the most recent evidence in the literature to (1) review the different types of technological tools used for self-management of sickle cell disease, (2) discover and describe what self-management activities these tools were used for, and (3) assess the efficacy of these technologies in self-management.

**Methods:**

We reviewed literature published between 1995 and 2016 with no language limits. We searched MEDLINE, EMBASE, CINAHL, PsycINFO, and other sources. We followed the Preferred Reporting Items for Systematic Reviews and Meta-Analyses guidelines. Two independent reviewers screened titles and abstracts, assessed full-text articles, and extracted data from articles that met inclusion criteria. Eligible studies were original research articles that included texting, mobile phone–based apps, or other eHealth interventions designed to improve self-management in pediatric and adult patients with sickle cell disease.

**Results:**

Of 1680 citations, 16 articles met all predefined criteria with a total of 747 study participants. Interventions were text messaging (4/16, 25%), native mobile apps (3/16, 19%), Web-based apps (5/16, 31%), mobile directly observed therapy (2/16, 13%), internet-delivered cognitive behavioral therapy (2/16, 13%), electronic pill bottle (1/16, 6%), or interactive gamification (2/16, 13%). Interventions targeted monitoring or improvement of medication adherence (5/16, 31%); self-management, pain reporting, and symptom reporting (7/16, 44%); stress, coping, sleep, and daily activities reporting (4/16, 25%); cognitive training for memory (1/16, 6%); sickle cell disease and reproductive health knowledge (5/16, 31%); cognitive behavioral therapy (2/16, 13%); and guided relaxation interventions (1/16, 6%). Most studies (11/16, 69%) included older children or adolescents (mean or median age 10-17 years; 11/16, 69%) and 5 included young adults (≥18 years old) (5/16, 31%). Sample size ranged from 11 to 236, with a median of 21 per study: <20 in 6 (38%), ≥20 to <50 in 6 (38%), and >50 participants in 4 studies (25%). Most reported improvement in self-management–related outcomes (15/16, 94%), as well as high satisfaction and acceptability of different study interventions (10/16, 63%).

**Conclusions:**

Our systematic review identified eHealth interventions measuring a variety of outcomes, which showed improvement in multiple components of self-management of sickle cell disease. Despite the promising feasibility and acceptability of eHealth interventions in improving self-management of sickle cell disease, the evidence overall is modest. Future eHealth intervention studies are needed to evaluate their efficacy, effectiveness, and cost effectiveness in promoting self-management in patients with sickle cell disease using rigorous methods and theoretical frameworks with clearly defined clinical outcomes.

## Introduction

### Background

Sickle cell disease (SCD) is an inherited blood disorder that affects more than 5 million individuals worldwide, and about 250,000 babies with SCD are born every year, mainly in Africa [1]. SCD affects over 100,000 Americans, mainly African Americans, many of whom are of lower socioeconomic status [2-5]. Advancements in treatment over the past few decades have changed the course of SCD from a condition of childhood to a chronic disease of adulthood [6]. Individuals with SCD are subject to acute and chronic complications, including vaso-occlusive pain crisis, acute chest syndrome, stroke, cognitive dysfunction, and end-organ damage to the liver, spleen, and kidneys, substantially reducing health-related quality of life and leading to early death [7,8]. Management of these SCD complications has a significant impact on health care utilization in the United States, with over 230,000 emergency room visits per year with an annual health expenditures of US $1.5 billion [9,10].

As part of the chronic care model [11], creating the informed, activated patient, along with the proactive care team, can lead to better health outcomes. One essential component of the informed, activated patient is the concept of self-management. Self-management has been referred to as the individual’s ability to manage the symptoms, treatment, physical and psychological consequences, and lifestyle changes inherent in living with a chronic condition [12]. SCD patients with more self-management skills can better manage their illness and potentially improve their health outcomes. Self-management skills are key for SCD patients as they encounter challenges related to managing their illness, such as pain management, adequate hydration, balanced nutrition, clinic attendance, and adherence to medication regimens, especially after they transition from pediatric to adult care settings. In particular, medication adherence is an important component of self-management. SCD patients with more reported adherence barriers and negative perceptions of hydroxyurea or SCD reported lower adherence rates and worse health-related quality of life scores [13-15]. In addition, many SCD patients were interested in having mobile apps with up-to-date clinical care guidelines that can improve understanding of the importance of self-management [16] and apps with features to improve their disease self-management [17]. Different techniques have been used to foster greater self-management and involve nontechnological solutions (eg, face-to-face or paper-based interventions). Over the last two decades, however, technological solutions, especially using the internet and mobile phones, to improve self-management have gained momentum with the wide access to mobile devices. These solutions, particularly electronic health (eHealth) interventions, potentially provide the benefit of greater acceptance and dissemination. eHealth has been defined as “an emerging field in the intersection of medical informatics, public health and business, referring to health services and information delivered or enhanced through the internet and related technologies” [18].

Access to personal technology is ubiquitous, and technological solutions are becoming a part of the way health care is delivered. People are more frequently using technology for their health [19,20], and there are government mandates, including meaningful use in the United States [21], that require health care providers to use technology in the care of their patients. Moreover, enhanced patient activation, as well as engagement in medical care and shared decision making, has been associated with improved health outcomes [22-24]. eHealth interventions have been shown to improve patient activation and engagement [25-29], making them a possible solution to improve outcomes. In addition, individuals with SCD and their families want to use technologies for their health [17,30,31]. While some eHealth technological interventions are being created and tested in SCD, these interventions have not been sufficiently evaluated in the few existing studies. Furthermore, a discussion about what interventions exist, how efficacious they are, and how they are being used to improve disease self-management is missing in this population.

In other chronic diseases, such as diabetes and asthma, a growing body of evidence has described improvements in self-care through the use of technological interventions [32-34]. Additionally, recent systematic reviews showed promising data to support the overall feasibility, acceptability, and efficacy of mobile interventions in improving health outcomes [35-40], although cost effectiveness remains unclear [41]. However, to the best of our knowledge, there has not been a systematic review of technological interventions used to improve self-management in the care of SCD. Further, evidence is growing to support the benefits of using mobile interventions to improve self-management in patients with chronic health conditions living in low- and middle-income countries [42-49]. Given the broad access to personal technology, as well as the high prevalence of SCD in many African countries, developing and implementing evidence-based mobile interventions could provide an opportunity to improve self-management skills and health outcomes in this population.

### Objectives

To understand how eHealth technology has been used to increase self-management of SCD and to guide future research, we performed a systematic review of the literature with the following objectives: (1) to review the different types of technological tools used for self-management of SCD, (2) to discover and describe what self-management activities these tools were used for, and (3) to assess the efficacy of these technologies in self-management. We conclude with gaps that will need to be addressed in future research.

## Methods

### Literature Search

This systematic review covered literature published between 1995 and 2016 with no language limits. The search strategy looked for all articles on texting, phones, mobile phone apps, portable software, and other eHealth interventions combined with sickle cell search terms. We intentionally used the Boolean operator OR instead of AND to capture the most comprehensive set of articles possible to which we applied our eligibility criteria. In brief, a medical librarian conducted the literature search in the following sources from inception to August 30, 2016: MEDLINE (through PubMed), EMBASE, Web of Science, Cochrane Central Register of Controlled Trials, CINAHL, PsycINFO, Engineering Village, and ClinicalTrials.gov databases. After the initial search, the results of the search were limited to articles published from 1995 to the date of the search on November 22, 2016. Our search strategy began with the MEDLINE search and was translated to the appropriate syntax for each of the other databases. In addition, we hand searched related themes. Multimedia Appendix 1 shows the detailed search strategies. We followed the Preferred Reporting Items for Systematic Reviews and Meta-Analyses (PRISMA) guidelines in the reporting of evidence across the studies reviewed herein (Multimedia Appendix 2) [50]. Two independent reviewers (SMB and RMC) assessed abstracts and articles against the eligibility criteria and critically appraised the methodological quality using established criteria from the Centre for Evidence-based Medicine [51]. Disagreements were resolved by discussion or consultation with a colleague, if needed.

### Eligibility Criteria

Eligible studies were original research articles reporting randomized controlled trials (RCTs), quasi-experimental studies, or pilot pre-post studies of texting, mobile phone–based apps, or other eHealth interventions designed to improve self-management in pediatric and adult patients with SCD. To be included in this review, the studies had to report at least one primary or secondary outcome related to self-management behavior, such as medication adherence, pain management, or education. We excluded studies focused on physicians or providers, or other aspects of SCD care other than self-management.

### Data Synthesis

We used a standardized form for data extraction. Data items in the extraction form were the following: first author’s name; publication year; country; aim of the study; participants’ age and sex; study design and setting; sample size; selection criteria; duration of intervention and follow-up; retention rate; components of the study intervention (texting, mobile phone apps, or other eHealth interventions) and comparator (if applicable); self-management measures and outcomes; other related outcome; and theoretical framework.

## Results

### Literature Search

**Figure 1:**
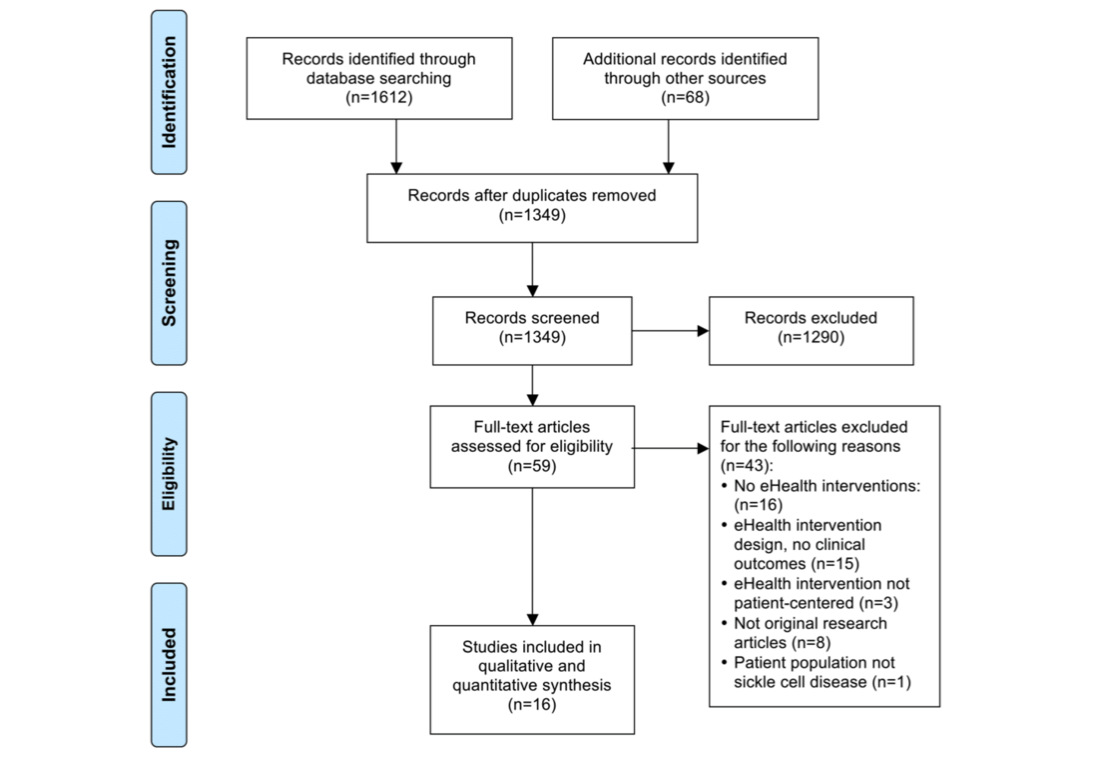
Preferred Reporting Items for Systematic Reviews and Meta-Analyses (PRISMA) flow diagram.

We retrieved a total of 1680 citations: 1612 identified through searching different databases and 68 through other resources. After we removed duplicates, 1349 original articles remained (Figure 1). Two authors (SMB and RMC) independently screened the article titles and abstracts of the 1349 records against the inclusion criteria, and a total of 59 met all predefined inclusion criteria. The same 2 authors (SMB and RMC) then independently reviewed the full text of these articles in detail against the exclusion criteria and excluded 43 articles. Finally, 16 articles met all predefined criteria to be included in this review with a total of 747 study participants [52-67]. We did not identify any non-English articles that met our inclusion criteria. Figure 1 shows the study PRISMA flowchart and documents the reasons for exclusion of full-text articles.

### Description of Included Studies

Multimedia Appendix 3 [52-67] summarizes the studies’ characteristics. The aims of the interventions were (1) monitoring or improvement, or both, of medication adherence, including hydroxyurea [53,54,58,63], iron chelation [61], or asthma medications [63]; (2) self-management [52,59,60,62, 64,66]; (3) pain and symptom reporting [52,55,59,60,62,64]; (4) stress, coping, sleep, and daily activities reporting [55,59,62,64]; (5) cognitive training for memory [57]; (6) disease education to improve SCD and [56,61,65-67] reproductive health knowledge [56,65]; and (7) cognitive behavioral therapy [62,64] and guided relaxation interventions [55]. All studies were performed in the United States [52-67]. Enrollment was mainly from clinics [52-58,60-66], as well as inpatients [67], the public [65], online networks [65], home [56,65], or community-based organizations [56,59,65]. All studies were conducted in the outpatient setting [52-66], except for 1 study conducted in the inpatient setting [67]. Most studies included older children or adolescents (mean or median age 10-17 years) [52-54,57-59,61,62,64,66,67], 5 studies included young adults (≥18 years old) [55,56,60,63,65], and 2 studies allowed parents to participate [54,58]. None of the included studies involved potential users, patients, or parents in the development of the intervention before it was tested.

Sample size ranged from 11 to 236, with a median of 21 per study; 6 enrolled fewer than 20 participants [53,57,58,60-62], 6 had 20 to 50 participants [52,55,56,63,64,66], and 4 enrolled more than 50 participants [54,59,65,67].

### Description of Study Methodologies

Study design varied in the included studies: 7 were pre-post pilot or feasibility trials [53,56-58,60,61,66], 5 were RCTs [55,62-65], 2 were single-arm observational studies [52,59], 1 was a quasi-experimental study [67], and 1 was a retrospective study [54]. Of the RCTs, 3 were nonblinded [62-64], 1 was single-blinded (participants) [65], and 1 was double-blinded [55]. Details of allocation concealment and study blinding were not adequately reported. None of the RCTs used intention-to-treat analysis. Retention rates differed across studies: less than 80% in 4 studies [52,60,64,67], from 80% to less than 100% in 6 studies [55,58,61-63,65], 100% in 1 study [53], and not reported in 5 studies [54,56,59,66,67]. The duration of the intervention ranged from 3 days to 12 months as follows: 3 months or less [55-57,60,62,63,67], more than 3 to 6 months or less [53,58,59,61,64], or more than 6 up to 12 months [52,54]. A total of 3 studies implemented a reward system to enhance participant engagement during the study intervention [52,53,67], and 1 study assessed the sustainability of the intervention effects with 3-month follow-up after the completion of the active intervention [61]. Additionally, 6 studies were informed by a clear theoretical framework for their intervention effects, as follows: health belief model [63]; theory-based game design [66]; gate control theory [55]; transactional stress model [67]; coping theoretical model [67]; theory of reasoned action [56,65]; and Kolb experiential learning theory [56,65].

### Description of eHealth Interventions

Interventions included text messaging [52-54,59,63], native mobile apps [57,60,61], Web-based apps [52,55,56,59,65], mobile directly observed therapy [53,61], internet-delivered cognitive behavioral therapy [62,64], electronic pill bottle [58], or interactive gamification [66,67]. A total of 4 studies indicated regular or mobile phone ownership or access as a requirement for study participation [52-54,63], while loaner phones [59,62,64], loaner tablets [55-57,65], or both [60,61] were available in other studies. Multimedia Appendix 4 [52-67] summarizes the various intervention components for all included studies.

### Intervention Effects on Outcomes

Study outcomes varied across studies, including medication adherence [53,54,58,61,63], disease knowledge [56,61,65-67], reproductive health knowledge [56,65], pain or symptom reporting completion rates [52,55,59,60,62,64], health care utilization [54,59], total opioid use [55], self-management skills [52,59,62,64,66], and coping and social support [67]. Almost all studies (15/16, 94%) reported improvement in self-management outcomes [52-57,59-67]. Most (10/16, 63%) reported high satisfaction and acceptability of different study interventions [52,53,55,56,59-62,65,67], while 6 studies did not report on these outcomes. Table 1 summarizes the main outcomes for all included studies.

**Table 1:** Summary of the main outcomes for all included studies with eHealth interventions.

Study	Main outcome
Jonassaint, 2015 [60]	High correlation between paper and electronic (SMART^a^ app) pain measurements; high association between pain severity and pain intensity using SMART app; daily entries using SMART app entries: 86% in week 1 and 58% in week 4; higher rates of daily entries with iPads and patients >35 years old; high usability and acceptability as a tool to monitor daily pain and other symptoms.
Hardy, 2016 [57]	Average number of completed sessions was 15.83 (SD 7.73); participants with higher completion rates were female and had lower pain scores; participants who completed scheduled intervention (Cogmed) sessions had improved verbal working memory, as well as visuospatial short-term and working memories.
Leonard, 2017 [61]	Participants tracked their medication usage about 80% at 30- and 90-day follow-up; high disease knowledge retention; adherence to iron chelation improved at 6-month follow-up as measured by serum ferritin levels and medication possession ratio; high satisfaction and acceptability as a tool to monitor medication adherence.
Creary, 2014 [53]	Adherence to hydroxyurea improved at 6-month follow-up as measured by fetal hemoglobin, mean corpuscular volume, and medication possession ratio; high satisfaction with electronic directly observed therapy (e-DOT) as a tool to monitor medication adherence; e-DOT needed 5 minutes or less to complete every day.
Estepp, 2014 [54]	Adherence to hydroxyurea improved as measured by laboratory markers (hemoglobin, fetal hemoglobin, mean corpuscular volume, absolute reticulocyte counts bilirubin levels); adherence to hydroxyurea improved as measured by medication possession ratio; no noticeable change in the number of hospitalizations or emergency room visits.
Pernell, 2017 [63]	Response rate to daily messages varied and was overall <50%; medication adherence self-report improved in the intervention group, but not in controls; asthma control test scores improved in the intervention group in adults, but not children.
Inoue, 2016 [58]	Hydroxyurea adherence rates were 85% as measured by either the electronic pill bottle GlowCap or medication possession ratio; laboratory markers of hydroxyurea adherence varied; a few technical challenges were also reported.
McClellan, 2009 [62]	Participants practiced I-CBT^b^ coping skills with different frequencies; self-report practice rates were higher in older and male participants; high satisfaction as a tool for pain, sleep, coping, and daily activities reporting.
Schatz, 2015 [64]	Number of active psychological coping attempts increased with the intervention; reduction in pain scores when participants used I-CBT skills the day before for higher pain; no association between participants’ skill use and functional activity.
Ezenwa, 2016 [55]	Intervention participants had significant reduction in current pain and stress levels; intervention participants had significant reduction in 2-week pain, but not stress intensity; no differences in total opioid use; high satisfaction with the tablet-based guided relaxation intervention to reduce pain.
Bakshi, 2017 [52]	Pain was reported most of the study days (76%); 50th and 90th percentiles of maximum daily pain directly correlated positively with mean maximum daily pain; proportion of pain-free days inversely correlated with mean maximum daily pain; highest pain diary completion rates were in first 30 days, which decreased over time; high satisfaction with momentary pain reporting and communication with medical team.
Jacob, 2013 [59]	Many children and adolescents reported mild to severe pain at home that did not require further evaluation by a health care professional; reported symptoms varied, including tiredness/fatigue, headache, yellowing of the eyes, and respiratory and musculoskeletal symptoms; higher pain scores were associated with shorter sleep duration and lower sleep quality; having previous history of SCD^c^-related events, symptoms, and negative thoughts was associated with reporting more frequent and higher-intensity pain; no differences in health care utilization (eg, emergency room visits or hospitalizations); high usability and acceptability as a tool to monitor daily pain and other symptoms.
Gallo, 2014 [56]	Intervention participants reported increased disease and reproductive knowledge scores; high acceptability of the CHOICES intervention; participants provided constructive feedback (eg, content, visualization, animation).
Wilkie, 2013 [65]	Intervention participants reported increased disease and reproductive knowledge scores; intervention participants were more likely to report a parenting plan to avoid SCD or SCD and sickle cell trait; there was an intervention effect on participants’ parenting intention and planned behavior.
Hazzard, 2002 [67]	Participants’ knowledge about SCD and asthma increased; participants reported more positive perceptions of peer support and less negative coping.
Yoon, 2007 [66]	Participants’ SCD knowledge and confidence levels increased significantly.

^a^SMART: Sickle cell disease Mobile Application to Record symptoms via Technology.

^b^I-CBT: internet-delivered cognitive behavioral therapy.

^c^SCD: sickle cell disease.

## Discussion

### Principal Findings

eHealth is increasingly being used for self-management of a variety of chronic diseases, including asthma, diabetes, and hypertension [46], as well as SCD. Despite systematic reviews describing eHealth use in other chronic diseases, to our knowledge, this is the first systematic review of eHealth for self-management of SCD. Our review demonstrates a range of eHealth interventions measuring a variety of outcomes, which showed improvement in multiple components of self-management of SCD. We also showed that few eHealth studies in SCD used rigorous methods, were grounded in theoretical frameworks, or measured clinical outcomes. This review describes the promise of eHealth to improve the care of individuals with SCD; however, many areas of future research can help demonstrate the usefulness of eHealth in this population.

Most studies were in children and adolescents with SCD. Many of these studies focused on adherence to medications such as hydroxyurea or iron chelation. Other systematic reviews looked at medication adherence using eHealth [68-73], with 1 of them looking specifically at the adolescent population [39]. Most of the studies in pediatric SCD had small sample sizes, and 1 was an RCT. These studies confirmed improvement in medication adherence in the participants receiving eHealth interventions. There is significant promise for improving medication adherence using eHealth in SCD, but larger, more methodologically rigorous studies demonstrating an improved effect are needed. Most of the other studies in our review focused on pain management and coping strategies in children. These studies also demonstrated good adherence to pain diaries and improved coping. One systematic review looked at the use of eHealth in pain [74], but the studies in this review were primarily in middle-aged participants. Another review described that studies in eHealth for pain in children are lacking [75]. The studies in this review exhibited the potential for eHealth interventions to improve self-management of pain in children with painful chronic diseases. Further, in our review, only 2 studies allowed caregivers to participate. Caregivers are an essential component of the care of the child, and more studies are needed to evaluate the use of eHealth in the parent-and-child dynamic to better understand optimal use of eHealth for both parts of this dynamic.

Intervention design did not vary according to patient characteristics, such as age, educational level, or other SCD-specific factors, which would be important for future intervention studies to consider as a strategy to optimize behavior change and long-term engagement. Additionally, 1 study was conducted in an inpatient setting, where management is more controlled by the health care system, whereas the goal of self-management interventions is to engage and empower patients in the outpatient setting with their day-to-day activities. In the outpatient setting, the health care system has less control, and the patient has more responsibility for disease management. More research is needed to evaluate the value of starting behavior change in the inpatient setting that could help to maintain intervention effects in the outpatient setting.

Relatively few studies evaluated eHealth in adults with SCD. This is in contrast to the number of systematic reviews of the use of eHealth in adults with other chronic diseases [33,46,76]. Most of the studies in adults with SCD focused on pain or knowledge about reproductive health in this age range, with only 1 study focused on medication adherence. However, the overall number of studies was fewer, and they were less concentrated on medication adherence, than the studies focused on eHealth use in children with SCD. More studies are needed in adults with SCD to demonstrate improvement in other components of care, including medication adherence for other medications, coping strategies, and clinic appointment adherence. Interestingly, half of the studies in this group were RCTs, which was more than those conducted in children and adolescents.

Studies in other chronic diseases measured outcomes unexplored in SCD. Multiple systematic reviews of eHealth in other chronic diseases saw improvements in clinical outcomes such as glycemic, blood pressure, and lipid level control [46,77]. None of the studies in our review evaluated the effect of eHealth interventions on outcomes for SCD, such as episodes of acute chest syndrome, strokes, or vaso-occlusive episodes of pain requiring emergency room visits or hospitalizations. Some of the pain studies in our review evaluated days and severity of pain, but these studies did not measure those pain episodes that resulted in health care utilization. Other reviews demonstrated improvement in clinic appointment adherence with eHealth interventions [76]. While there were preliminary studies in SCD describing an eHealth app to help with clinic appointments [78], there were no formal evaluation studies to demonstrate improvement in clinic attendance. Other studies looked at improving patient activation using eHealth in other diseases [79]; Risling and colleagues’ review was primarily about patient portals that improve activation. Our review did not identify any studies that demonstrated improvement in patient activation in SCD, and we found no studies of patient portals as the eHealth intervention. Expanding the range of outcomes measured in the use of eHealth for self-management of SCD is a potential area for future research.

While we included articles that reported RCTs, quasi-experimental studies, or pilot pre-post studies, many preliminary studies and clinical trials are underway to develop and evaluate the next set of eHealth tools. These studies include preliminary needs assessments [17,80-83], processes for development of a tool [83,84], prototypes [84-87], pilot feasibility studies [31,83,88,89], and ongoing clinical trials [90,91] for eHealth interventions. A reason there may be fewer interventions published about SCD could be related to health information technology disparities with other diseases such as cystic fibrosis [92]. There is promise that mobile health technologies can help bridge this digital divide. With the increased uptake of mobile technology use and the number of preliminary studies in SCD, this is a prime area for future research. In addition, many studies have discussed improvement in self-management using eHealth in low- and middle-income countries [42-49]. While our review of the literature saw a paucity of studies from these countries, there is significant potential for eHealth to improve self-management of SCD in Africa, where the burden of SCD is much greater than in higher-income countries and the improvement in self-management with eHealth has been demonstrated [42-49]. Despite the promise of bridging the digital divide, lack of access to the mobile data plans required to deliver eHealth interventions could be a potential barrier to the wide dissemination of such interventions in middle- and low-income countries.

Despite the potential of eHealth to improve self-management of SCD, the SCD community and their health care providers need to exercise caution when using eHealth interventions. Many eHealth apps are available in online stores, but their evaluation is lacking. As seen in a systematic review of pain apps, little research of the many apps available has been published [93]. Most apps have not been studied and are not regulated by governmental bodies. Use of these apps can come with significant risk to the accuracy of information delivered, as well as data privacy and security risks. The accuracy of the information included in different health apps is another major risk for users, and the associated costs with purchasing these apps could be a burden for many patients and a potential barrier to uptake. Evaluation of eHealth interventions as they are made available will be crucial in aiding providers and patients to choose eHealth tools that will be safe and effective in improving the care of people with chronic diseases.

### Strengths and Limitations

Our systematic review has a number of strengths. First, in our review, we followed the recommendations for rigorous systematic review methodology [50,51,94-96]. Second, we conducted a review with a highly sensitive search strategy, guided by a medical information specialist, with no language restrictions so as to minimize publication bias and identify the largest possible number of relevant studies. Our search also included published systematic reviews, clinical trial registries, and various electronic databases. Third, although our search was limited to studies published since 1995, we identified no eligible studies before 2005, and therefore we believe that the possibility of missing earlier studies is very small. Fourth, 2 authors completed the review process independently at all stages of the systematic review.

Some potential methodological limitations of our systematic review warrant discussion. First, similar to any other systematic literature review, although we planned our search criteria to be as comprehensive as possible, the possibility of missing a few relevant articles cannot be excluded. Second, to identify the strongest available evidence, we included articles that were published in peer-reviewed journals, and therefore there could be a publication bias with the tendency to report positive study results [97]. Third, the study sample sizes and ages, and the definition of adherence to preventive behaviors and other related outcomes varied. These limitations prohibited a meta-analysis from being performed [98]. Fourth, some of the included studies had relatively small sample sizes.

### Conclusions

Our systematic review is, to the best of our knowledge, the first to evaluate eHealth for self-management in pediatric and adult patients with SCD. We identified several eHealth interventions measuring a variety of outcomes, which showed improvement in multiple components of self-management of SCD. Despite the promising feasibility and acceptability of eHealth interventions in improving self-management of SCD, the evidence overall is modest. However, with the increased access to mobile technology, eHealth interventions have great potential to improve health outcomes in patients with SCD, as well as other chronic diseases. Future eHealth intervention studies are needed to evaluate their efficacy, effectiveness, and cost effectiveness in promoting self-management in patients with SCD using rigorous methods and theoretical frameworks with clearly defined clinical outcomes. This review describes the promise of eHealth to improve self-management in individuals with SCD; however, there are many areas of future research that can help demonstrate their usefulness in this population.

## Appendix

Multimedia Appendix 1 Search strategies.

Multimedia Appendix 2 Preferred Reporting Items for Systematic Reviews and Meta-Analyses (PRISMA) checklist.

Multimedia Appendix 3 Summary of included studies focused on eHealth interventions.

Multimedia Appendix 4 Summary of the components of eHealth interventions for all included studies.

## Abbreviations

eHealth: electronic health
PRISMA: Preferred Reporting Items for Systematic Reviews and Meta-Analyses
RCT: randomized controlled trial
SCD: sickle cell disease
