# Supplementary material for: Patient-Centered eHealth Interventions for Children, Adolescents, and Adults With Sickle Cell Disease: Systematic Review
Source: J Med Internet Res. 2018 Jul 19;20(7):e10940. doi: 10.2196/10940 (PMC6072976; doi:10.2196/10940)
Supplement: Multimedia Appendix 1 [file jmir_v20i7e10940_app1.pdf]

Appendix I – Search Strategies

Database Searched	Date Searched	Results
MEDLINE via PubMed, 1946 to 11/22/16	11/22/16	238
EMBASE (embase.com) 1946 to 11/22/16	11/22/16	602
Science Citation Index Expanded (SCI-EXPANDED) 1900 to 11/22/16; Social Sciences Citation Index (SSCI) –2000 to 11/22/16;<br>Conference Proceedings Citation Index- Science (CPCI-S) 1990 to 11/22/16;<br>Conference Proceedings Citation Index- Social Science & Humanities (CPCI-SSH) 1990 to 11/22/16<br>(Web of Science)	11/22/16	154
Cochrane Database of Controlled Trials: Issue 11 of 12, November 2016 (Wiley)	11/22/16	302
CINAHL (EBSCO) 1961 to 11/22/16	11/22/16	298
PsycINFO (EBSCO) 1967 to 11/22/16	11/22/16	18
Engineering Village (Compendex, Elsevier) 1970 to 11/22/16	11/22/16	32
ClinicalTrials.gov	11/22/16	36
Total		1680
After de-duplication		1350

Search Approach

We searched PubMed MEDLINE, Embase, Web of Science, Cochrane Database of Controlled Trials, CINAHL, PsycINFO, Engineering Village, and ClinicalTrials.gov databases from inception to 8/30/16. After the initial search, we then limited the results to articles published from 1995 to the date of the search on 11/22/16. We began with the MEDLINE search and translated to the appropriate syntax for each of the other databases. See the search strategies for each of the databases are below.

1. MEDLINE via PubMed

(((“Anemia, Sickle Cell”[Mesh] OR “sickle cell”[tw] OR “haemoglobin S”[tw] OR “hemoglobin S”[tw]))) AND (“mobile health”[tw] OR “mhealth”[tw] OR “ehealth”[tw] OR “m-health”[tw] OR “e-health”[tw] OR “mcare”[tw] OR “Cell Phones”[Mesh] OR “cellphone”[tw] OR “cellphones”[tw] OR “cell phones”[tw] OR “cell phone” OR “cellular phone”[tw] OR “cellular phones”[tw] OR “cellular telephone”[tw] OR “cellular telephones”[tw] OR “mobile phone”[tw] OR “mobile phones”[tw] OR “mobile telephone”[tw] OR “mobile telephones”[tw] OR “iphone”[tw] OR “iphones”[tw] OR “Microcomputers”[Mesh] OR “microcomputer”[tw] OR “microcomputers”[tw] OR

“handheld computer”[tw] OR “handheld computers”[tw] OR “hand held computer”[tw] OR “hand held computers”[tw] OR “ipad”[tw] OR “ipads”[tw] OR “pda”[tw] OR “pdas”[tw] OR “personal digital assistant”[tw] OR “personal digital assistants”[tw] OR “blackberry”[tw] OR “android”[tw] OR “androids”[tw] OR “smartphone”[tw] OR “smartphones”[tw] OR “smart phone”[tw] OR “smart phones”[tw] OR “tablet”[tw] OR “apps”[tw] OR “app”[tw] OR “mobile application”[tw] OR “mobile applications”[tw] OR “mobile communication”[tw] OR “mobile communications”[tw] OR “mobile technology”[tw] OR “mobile technologies”[tw] OR “mobile game”[tw] OR “mobile games”[tw] OR “Internet”[Mesh] OR “internet”[tw] OR “Computer Simulation”[Mesh] OR “computer simulation”[tw] OR “Electronic Mail”[Mesh] OR “email”[tw] OR “emails”[tw] OR “emailing”[tw] OR “e-mail”[tw] OR “e-mails”[tw] OR “e-mailing”[tw] OR “electronic mail”[tw] OR “Reminder Systems”[Mesh] OR “reminder systems”[tw] OR “reminder system”[tw] OR “Wireless Technology”[Mesh] OR “wireless technology”[tw] OR “wireless technologies”[tw] OR “wireless communication”[tw] OR “wireless communications”[tw] OR “Software”[Mesh] OR “software”[tw] OR “Video Recording”[Mesh] OR “video recording”[tw] OR “video recordings”[tw] OR “Text Messaging”[Mesh] OR “text message”[tw] OR “text messaging”[tw] OR “texting”[tw] OR “text”[tw] OR “texts”[tw] OR “SMS”[tw] OR “short message service”[tw] OR “text messages”[tw])

2. Embase

(‘mobile phone’/exp OR ‘mobile phones’ OR ‘smart phone’ OR ‘smart phones’ OR ‘cell phone’ OR ‘cell phones’ OR ‘cellphone’ OR ‘cellphones’ OR ‘cellular phone’ OR ‘cellular phones’ OR ‘cellular telephone’ OR ‘cellular telephones’ OR ‘mobile telephone’ OR ‘mobile telephones’ OR ‘iphone’ OR ‘ipad’ OR ‘iphones’ OR ‘ipads’ OR ‘mobile application’ OR ‘mobile applications’ OR ‘mobile app’ OR ‘mobile apps’ OR ‘portable software app’ OR ‘portable software application’ OR ‘portable software apps’ OR ‘personal digital assistant’ OR ‘blackberry’ OR ‘android’ OR ‘androids’ OR ‘hand held computer’ OR ‘handheld computer’ OR ‘hand held computers’ OR ‘handheld computers’ OR ‘smartphone’ OR ‘smart phone’ OR ‘smartphones’ OR ‘smart phones’ ‘mobile communication’ OR ‘mobile communications’ OR ‘mobile technology’ OR ‘mobile technologies’ OR ‘tablet’ OR ‘mobile games’ OR ‘microcomputer’ OR ‘microcomputers’ OR ‘mhealth’ OR ‘mcare’ OR ‘ehealth’ OR ‘m-health’ OR ‘e-health’ OR ‘internet’/de OR ‘internet’ OR ‘computer simulation’/de OR ‘computer simulation’ OR ‘e-mail’/de OR ‘email’ OR ‘e-mail’ OR ‘emailing’ OR ‘e-mailing’ OR ‘electronic mail’ OR ‘reminder system’/de OR ‘reminder system’ OR ‘reminder systems’ OR ‘wireless communication’/de OR ‘wireless communication’ OR ‘wireless communications’ OR ‘wireless technology’ OR ‘wireless technologies’ OR ‘videorecording’/de OR ‘video recording’ OR ‘video recordings’ OR ‘text messaging’/de OR ‘text messaging’ OR ‘text message’ OR ‘text messages’ OR ‘texting’ OR ‘text’ OR ‘texts’ OR ‘SMS’ OR ‘short message service’) AND (‘sickle cell anemia’/de OR ‘sickle cell’ OR ‘haemoglobin S’ OR ‘hemoglobin S’)

3. CENTRAL

#1 MeSH descriptor: [Anemia, Sickle Cell] explode all trees
#2 "sickle cell" or "haemoglobin S" or "hemoglobin S"
#3 MeSH descriptor: [Cell Phones] explode all trees
#4 MeSH descriptor: [Microcomputers] explode all trees
#5 MeSH descriptor: [Internet] explode all trees
#6 MeSH descriptor: [Electronic Mail] explode all trees
#7 MeSH descriptor: [Reminder Systems] explode all trees
#8 MeSH descriptor: [Wireless Technology] explode all trees
#9 MeSH descriptor: [Video Recording] explode all trees
#10 MeSH descriptor: [Software] explode all trees
#11 MeSH descriptor: [Text Messaging] explode all trees
#12 "mobile health" or "mhealth" or "ehealth" or "m-health" or "e-health" or "mcare" or "cellphone" or "cellphones" or "cell phones" or "cell phone" or "cellular phone" or "cellular phones" or "cellular telephone" or "cellular telephones" or "mobile phone" or "mobile phones" or "mobile telephone" or "mobile telephones" or "iphone" or "iphones" or "microcomputer" or "microcomputers" or "handheld computer" or "handheld computers" or "hand held computer" or "hand held computers" or "ipad" or "ipads" or "pda" or "pdas" or "personal digital assistant" or "personal digital assistants" or "blackberry" or "android" or "androids" or "smartphone" or "smartphones" or "smart phone" or "smart phones" or "tablet" or "apps" or "app" or "mobile application" or "mobile applications" or "mobile communication" or "mobile communications" or "mobile technology" or "mobile technologies" or "mobile game" or "mobile games" or "internet" or "computer simulation" or "email" or "emails" or "emailing" or "e-mail" or "e-mails" or "e-mailing" or "electronic mail" or "reminder systems" or "reminder system" or "wireless technology" or "wireless technologies" or "wireless communication" or "wireless communications" or "software" or "video recording" or "video recordings" or "text message" or "text messaging" or "texting" or "text" or "texts" or "SMS" or "short message service" or "text messages"
#13 {or #1-#2}
#14 {or #3-#12}
#15 #13 and #14

4. CINAHL

((MH “cellular phone+”) OR (MH “computers, hand-held+”) OR TI “cell phone*” OR AB “cell phone*” OR TI “cellular phone*” OR AB “cellular phone*” OR TI “mobile phone*” OR AB “mobile phone*” OR TI “cellular telephone*” OR AB “cellular telephone*” OR TI “mobile telephone*” OR AB “mobile telephone*” OR TI iphone* OR AB iphone* OR TI ipad* OR AB ipad* OR TI cellphone* OR AB cellphone* OR TI pda* OR AB pda* OR TI “personal digital assistant*” OR AB “personal digital assistant*” OR TI tablet* OR AB tablet* OR TI smartphone* OR AB smartphone* OR TI “text messag*” OR AB “text messag*” OR TI texting OR AB texting OR TI “mobile application*” OR AB “mobile application*” OR TI app OR AB app OR TI apps OR AB apps OR TI “mobile game*” OR AB “mobile game*” OR TI blackberry OR AB blackberry OR TI android* OR AB android* OR TI “mobile health” OR AB “mobile health” OR TI mhealth OR AB mhealth OR TI

ehealth OR AB ehealth OR TI m-health OR AB m-health OR TI e-health OR AB e-health OR TI mcare OR AB mcare OR TI microcomputer* OR AB microcomputer* OR TI “handheld computer*” OR AB “handheld computer*” OR (MH “internet+”) OR TI internet OR AB internet OR TI “mobile technology” OR AB “mobile technology” OR (MH “electronic mail”) OR TI “electronic mail” OR AB “electronic mail” OR TI email* OR AB email* OR TI e-mail* OR AB e-mail* OR (MH “reminder systems”) OR TI “reminder system*” OR AB “reminder system*” OR (MH “computer simulation”+) OR TI “computer simulation” OR AB “computer simulation” OR (MH “wireless communication”) OR TI “wireless communication” OR AB “wireless communication” OR (MH “Software+”) OR TI software OR AB software OR (MH “videorecording”) OR TI videorecording* OR AB videorecording* OR TI video recording* OR AB video recording*) AND ((MH “anemia, sickle cell”) OR TI “sickle cell” OR AB “sickle cell” OR TI “haemoglobin S” OR AB “haemoglobin S” OR TI “haemoglobin S” OR AB “haemoglobin S”)

5. PsycINFO

((DE “cellular phone+”) OR (DE “computers, hand-held+”) OR TI “cell phone*” OR AB “cell phone*” OR TI “cellular phone*” OR AB “cellular phone*” OR TI “mobile phone*” OR AB “mobile phone*” OR TI “cellular telephone*” OR AB “cellular telephone*” OR TI “mobile telephone*” OR AB “mobile telephone*” OR TI iphone* OR AB iphone* OR TI ipad* OR AB ipad* OR TI cellphone* OR AB cellphone* OR TI pda* OR AB pda* OR TI “personal digital assistant*” OR AB “personal digital assistant*” OR TI tablet* OR AB tablet* OR TI smartphone* OR AB smartphone* OR TI “text messag*” OR AB “text messag*” OR TI texting OR AB texting OR TI “mobile application*” OR AB “mobile application*” OR TI app OR AB app OR TI apps OR AB apps OR TI “mobile game*” OR AB “mobile game*” OR TI blackberry OR AB blackberry OR TI android* OR AB android* OR TI “mobile health” OR AB “mobile health” OR TI mhealth OR AB mhealth OR TI ehealth OR AB ehealth OR TI m-health OR AB m-health OR TI e-health OR AB e-health OR TI mcare OR AB mcare OR TI microcomputer* OR AB microcomputer* OR TI “handheld computer*” OR AB “handheld computer*” OR (DE “internet+”) OR TI internet OR AB internet OR TI “mobile technology” OR AB “mobile technology” OR (DE “electronic mail”) OR TI “electronic mail” OR AB “electronic mail” OR TI email* OR AB email* OR TI e-mail* OR AB e-mail* OR (DE “reminder systems”) OR TI “reminder system*” OR AB “reminder system*” OR (DE “computer simulation”+) OR TI “computer simulation” OR AB “computer simulation” OR (DE “wireless communication”) OR TI “wireless communication” OR AB “wireless communication” OR (DE “Software+”) OR TI software OR AB software OR (DE “videorecording”) OR TI videorecording* OR AB videorecording* OR TI video recording* OR AB video recording*) AND ((DE “anemia, sickle cell”) OR TI “sickle cell” OR AB “sickle cell” OR TI “haemoglobin S” OR AB “haemoglobin S” OR TI “haemoglobin S” OR AB “haemoglobin S”)

6. Web of Science

TS= “cell* phone*” OR
TS= “cell* telephone*” OR
TS= smartphone* OR

TS= “smart phone*” OR
TS= “cellphone*” OR
TS= “tablet computer*” OR
TS= “tablet*” OR
TS= “mobile phone*” OR
TS= “mobile telephone*” OR
TS= iphone* OR
TS= ipad* OR
TS= blackberry OR
TS= android* OR
TS= “personal digital assistant*” OR
TS= “pda*” OR
TS= “mobile app*” OR
TS= “mobile game*” OR
TS= “app” OR
TS= “apps” OR
TS= “microcomputer*” OR
TS= “handheld computer*” OR
TS= “hand held computer*” OR
TS= “mobile communication*” OR
TS= “mobile technolog*” OR
TS= “internet” OR
TS= “computer simulation*” OR
TS= “electronic mail” OR
TS= “email*” OR
TS= “e-mail*” OR
TS= “reminder system*” OR
TS= “wireless communication*” OR
TS= “wireless technolog*” OR
TS= “software” OR
TS= “videorecording*” OR
TS= “video recording*” OR
TS= “text messag*” OR
TS= “texting” OR
TS= “text” OR
TS= “texts” OR
TS= “SMS” OR
TS= “short message service”
AND
TS= “sickle cell anemia” OR
TS= “sickle cell anaemia” OR
TS= “sickle cell disease” OR
TS= “hemoglobin S” OR
TS= “haemoglobin S” OR

Engineering Village
(“Sickle Cell” OR “hemoglobin S” OR “haemoglobin S”) AND (“mobile health” OR “mhealth” OR “ehealth” OR “m-health” OR “e-health” OR “mcare” OR “cellphone” OR “cellphones” OR “cell phones” OR “cell phone” OR “cellular phone” OR “cellular phones” OR “cellular telephone” OR “cellular telephones” OR “mobile phone” OR “mobile phones” OR “mobile telephone” OR “mobile telephones” OR “iphone” OR “iphones” OR “microcomputer” OR “microcomputers” OR “handheld computer” OR “handheld computers” OR “hand held computer” OR “hand held computers” OR “ipad” OR “ipads” OR “pda” OR “pdas” OR “personal digital assistant” OR “personal digital assistants” OR “blackberry” OR “android” OR “androids” OR “smartphone” OR “smartphones” OR “smart phone” OR “smart phones” OR “tablet” OR “apps” OR “app” OR “mobile application” OR “mobile applications” OR “mobile communication” OR “mobile communications” OR “mobile technology” OR “mobile technologies” OR “mobile game” OR “mobile games” OR “internet” OR “computer simulation” OR “email” OR “emails” OR “emailing” OR “e-mail” OR “e-mails” OR “e-mailing” OR “electronic mail” OR “reminder systems” OR “reminder system” OR “wireless technology” OR “wireless technologies” OR “wireless communication” OR “wireless communications” OR “software” OR “video recording” OR “video recordings” OR “text message” OR “text messaging” OR “texting” OR “text” OR “texts” OR “SMS” OR “short message service” OR “text messages”)

7. ClinicalTrials.gov

“Sickle Cell” AND “Mobile”
“Sickle Cell” AND “internet”
“Sickle Cell” AND “texting”
