# Supplementary material for: Patient-Centered eHealth Interventions for Children, Adolescents, and Adults With Sickle Cell Disease: Systematic Review
Source: J Med Internet Res. 2018 Jul 19;20(7):e10940. doi: 10.2196/10940 (PMC6072976; doi:10.2196/10940)
Supplement: Multimedia Appendix 3 [file jmir_v20i7e10940_app3.pdf]

Summary of included studies focused on eHealth interventions

**First author, date, reference**	**Intervention (study design)**	**Aim**	**Number of participants (sex)**	**Age in years**	**Follow up**	**Retention**
Jonassaint, 2015 [60]	Native app "SMART" (Pilot)	Pain and symptom reporting/managemen t	N=15 (40% female)	Median 29, range 16-54	4 weeks	70%
Hardy, 2016 [57]	Native app "Cogmed" (Pilot)	Cognitive training	N=12 (52% female)	Mean (SD) 11.4 (2.8)	5 weeks	50%
Leonard, 2017 [61]	Mobile-DOT "ITP" (Pilot)	Adherence to iron chelation	N=11 (64% female)	Median 11, range 8-21	6 months	90%
Creary, 2014 [53]	Mobile-DOT (Pilot)	Adherence to hydroxyurea	N=14 (50% female)	Mean (SD) 13.7 (6.3)	6 months	100%
Estepp, 2014 [54]	Text messaging "SIMON" (Retrospective)	Adherence to hydroxyurea	N=55 (53% female)	Median 13.9, range 12.1-16.1	12 months	N/A
Pernell, 2017 [63]	Text messaging "REDCap" (Pilot RCT)	Adherence to hydroxyurea and asthma medications	N=47; Control: 21, Intervention: 26; (49% female)	Median 20, range 3-59	2 months	83%
Inoue, 2016 [58]	Electronic pill bottle "Glow-Cap™" (Pilot)	Adherence to hydroxyurea	N=19 (47% females)	Median 6.5, range 2-21	11.5 months	85%
McClellan, 2009 [62]	I-CBT training "Motorola Q" (RCT)	Pain, sleep, coping and daily activities reporting	N=19; Control: 10, Intervention: 9; (68% female)	Mean (SD) 13.4 (2.9)	2 months	90%
Schatz, 2015 [64]	I-CBT training "Samsung Saga" (RCT)	Pain, coping and daily activities reporting	N=46; Control: 23, Intervention: 23; (59%	Mean (SD) 13 (2.5)	4 months	61%

			female)			
Ezenwa et al, 2016 [55]	Web-based "PAINReportIt" (RCT)	Pain, stress and coping reporting/management	N=27; Control: 12, Intervention: 15; (70% female)	Mean (SD) 31.7 (10.2)	2 weeks	89%
Bakshi, 2017 [52]	Web-based (Observational)	Pain	N=20 (75% female)	Median 16, range 13-21	7 months	50%
Jacob, 2013 [59]	Web-based (Observational)	Pain, symptoms, sleep and coping reporting	N=75 (54.3% females)	Mean (SD) 13 (1.9)	6 months	N/A
Gallo, 2014 [56]	Web-based "CHOICES" (Pilot pre-post)	SCD and reproductive health knowledge	N=20; Control: 10, Intervention: 10; (80% female)	Mean (SD) 27 (5)	4 weeks	N/A
Wilkie, 2013 [65]	Web-based "CHOICES" (RCT)	SCD and reproductive health knowledge	N=236; Control: 119; Intervention: 115 (65% female)	Mean (SD):<br>Control: 25.3 (5)<br>Intervention: 26.4 (4.9)	N/A	98%
Hazzard, 2002 [67]	Web-based "STARBRIGHT World" (Pilot)	SCD knowledge, coping and social support	N=47; Control: 29, Intervention: 18 (42% female)	Mean (SD) 11.7 (2.7)	3 days	N/A
Yoon, 2007 [66]	CD-ROM "Sickle Cell Slime-O-Rama Game" (Pilot)	SCD knowledge and self-management	N=22 (N/A)	Mean (SD) 10.9 (2.6)	N/A	N/A

I-CBT: Internet-delivered cognitive behavioral therapy; ITP: intensive training program; N/A: not available; RCT: randomized controlled trial; SCD: sickle cell disease; SD: standard deviation
