# Supplementary material for: Patient-Centered eHealth Interventions for Children, Adolescents, and Adults With Sickle Cell Disease: Systematic Review
Source: J Med Internet Res. 2018 Jul 19;20(7):e10940. doi: 10.2196/10940 (PMC6072976; doi:10.2196/10940)
Supplement: Multimedia Appendix 4 [file jmir_v20i7e10940_app4.pdf]

Summary of the components of different eHealth interventions for all included studies.

First author, date, reference	Intervention Components
Jonassaint, 2015 [60]	**SMART app**<br>• A native application for iOS (iPhone, iPod Touch, or an iPad)<br>• Pain intensity was assessed using an electronic VAS with a slider scale (0-10)<br>• Pain categories: ‘‘less’’, ‘‘same’’ or ‘‘worse’’ than typical pain, or ‘‘unbearable’’<br>• A ‘‘pain delta score’’ was automatically calculated with the pain difference from baseline<br>• An automated warning message with a suggested intervention were triggered if patients reported pain as “Unbearable” or “pain delta score” of ≥1.5 of baseline score<br>• Pain reported as one or more pain locations with or without other associated symptoms<br>• Patients reported pharmacological and non-pharmacological treatment strategies
Hardy, 2016 [57]	**Cogmed app**<br>• A home-based, computerized, interactive, audio-visual cognitive training program, installed via Internet download and compatible with Apple iOS and android-based tablet computers<br>• Series of game-like exercises with repeated practice of different tasks for visuospatial and auditory span memory<br>• Every exercise is adapted to the skill level of the individual user based on a trial-by-trial basis making tasks are always appropriately challenging<br><br>**Coaching**: A clinical psychologist served as trained intervention or “coach” who was available to provide support all participants by phone throughout the study
Leonard, 2017 [61]	**Intensive Training Program “ITP”**<br>• A provider-developed mobile app<br>• Downloaded directly onto participants’ iOS devices (iPhone, iPod, or iPad)<br>• Patients were given a loaner device, if they didn’t have a device<br>• The ITP mobile app has the following functions:<br>o Time-stamped monitoring that enables “real-time” adherence tracking<br>o Daily self-recorded videos (“selfies”) of medication administration<br>o Videos were uploaded to a secure encrypted HIPAA-compliant server

	o Remote access to provider-led disease education modules<br>o Patient support through reminder alert messages<br>o Behavioral reinforcement with provider messages and feedback about adherence every 7 to 10 days, based on the recorded “selfies”
Creary, 2014 [53]	**Mobile-DOT (**Multi-dimensional strategy)<br><br>• Alert reminders:<br>o Automated daily alerts to remind patients to take hydroxyurea<br>o Alert sent at time preferred by patients<br>o Alerts stopped when a video is submitted<br>o Up to four text messages and e-mail were sent daily<br>• Videos:<br>o Participants created daily videos of them taking hydroxyurea<br>o Videos submitted electronically to the secure study website<br>o Captured by smartphones or computers<br>o Included participants’ study ID<br>o Self-recorded videos for children ≥12 yrs, younger ones had assistance from parents<br>• Feedback:<br>o Submitted videos were reviewed by research team within 72 hours of receipt<br>o SMS and e-mail feedback was sent to participants if they missed 2 or more video submissions in a 30-day period<br>o Participants were called if they missed 3 or more video submissions in a 30-day period<br>o Positive reinforcement (at least two text messages or e-mails) was provided if they participants had adherence of 90% or more<br>• Incentives:<br>o If participants achieved 90% or more of adherence to hydroxyurea for each 30-day period, they would receive $1/day
Estepp, 2014 [54]	**Text messaging “SIMON”**<br><br>• Scheduled daily text message reminders for 12 months<br>• Customizable for content, frequency, and duration<br>• Participants created their own messages<br>• Changes in text messages regimen checked every 3-4 clinic visits<br>• Messages delivery was monitored (received and undelivered)<br>• Participants could optionally reply

	• Messages sent through web-based application
Pernell, 2017 [63]	**Text messaging "REDCap"**<br><br>• Two-way SMS medication reminders sent via Research Electronic Data Capture software<br>• Utilized the automated invitations feature in REDCap<br>• Daily and twice daily reminders based on prescribed medication regimens<br>• Three arms of the study (asthma, hydroxyurea, both asthma and hydroxyurea)<br>• The SMS reminders included the following text: "Did you take your [hydroxyurea] [asthma] medication [this morning] [this evening] [today]?"<br>• SMS reminders were customized based on participants' time preference of the day<br>• Separate reminders were sent if participants were on hydroxyurea and asthma medications<br>• Text messaging algorithm:<br>o Participants were prompted to reply '(1) yes' or '(0) no' as to whether they took their medication<br>o Participants that responded '(0) no' received an additional SMS reminder two hours later reminding them to take their medication<br>o If the participant did not respond to the initial text message, one additional text reminder with the same message was sent one hour later
Inoue, 2016 [58]	**Electronic Pill Bottle "GlowCap™**<br><br>• A trained research nurse gave detailed one-on-one instructions on how to use the device<br>• The device served as a medicine container as well as a signal transmitter<br>• Every time the cap is opened, an electronic signal or a time stamp was sent to the central database with the recorded date and time<br>• A separate reminder device that was plugged into a household electric outlet<br>• The device transmitted a reminder—first by flashing lights, then by sounding chimes if the patient is late opening the cap<br>• If 2 hours elapsed without the patient opening the cap of the device, the patient then received a telephone call reminder
McClellan, 2009 [62]; Schatz, 2015 [64]	**Internet-delivered Cognitive Behavioral Therapy "I-CBT"**<br><br>Participants were given either a Motorola Q or a Samsung Saga smartphone<br>A single-session training in CBT methods for pain management<br>CBT coping skills session:<br>o Psycho-education using participatory activities (Pain in sickle cell disease, Gate Control

	Theory, and active vs. passive coping)<br>o Rationale and demonstration of distraction, deep breathing, progressive muscle relaxation, and guided imagery using participatory activities<br>o Demonstration of deep breathing, progressive muscle relaxation, and guided imagery using the smartphone<br>o Demonstration of how to use the daily diary using the smartphone<br>o "Show What You Know Quiz"<br>Smartphone application:<br>o Daily diary application to complete ratings of pain and activity<br>o Skill practice/use application to access audio files for deep breathing, progressive muscle relaxation, and guided imagery<br>Written materials<br>o Pain management flowchart (Child's usual medications and how to take them; reminders to use distraction, deep breathing, progressive muscle relaxation, and guided imagery; and information about when to call the doctor (developed by hematologist)<br>o Coping skills handout (Brief rationale and instructions for skills; description of the child's favorite places for guided imagery; list of best activities the child generated for implementing distraction)<br>o Information about the technical aspects of the smartphone<br>Telephone calls<br>o Weekly check-in calls with study staff (once per week)<br>o Calls to address missing diaries (after 3 consecutive days of missing diaries)<br>o Technical support for smartphone issues (as requested)<br>Audio icons and files<br>o The audio files were unique to the study and developed by one of the coauthors<br>o Modeled after widely available scripts for these activities as found in numerous CBT manuals and commercial recordings for teaching these skills<br>o For example, a picture of a balloon would initiate the audio file for deep breathing, and the balloon icon had been incorporated into demonstrations and written materials provided at the training session
Ezenwa, 2016 [55]	**Guided Relaxation (GR) Intervention**<br>• A single 12-minute GR video clip administered at the baseline visit to determine its immediate effects on stress and pain<br>• Six video clips ranging from 2-20 minutes in length for home use

	• Video clips had similar content, longer ones had more repetitions of the same content<br>• Video clips were delivered on tablets that were programmed to prompt patients to track their stress and pain daily and allowed patients to select a video clip to watch<br>• GR patients were instructed to watch any of the six video clips at stress onset and as often as they desired, but at least once daily to obtain a minimal intervention dose<br>• Video clips were developed and validated in psycho-neuro-immunology studies<br>• Some of the active ingredients in the GR video clips include colourful smoke-like images that slowly change shapes against a dark background (images, therefore, do not represent any concept with potential negative connotations), a soothing female voice and slow-paced guided relaxation instructions<br>• Input from patient team members was used to modify these video clips to produce a patient-centered stress reduction GR intervention<br>• Patients recommended variety in the video lengths so that they could be more easily used throughout the day as needed
Bakshi, 2017 [52]	**e-Diary Web-based Intervention**<br>• A secure web-based application and database as a pain intensity diary<br>• Participants reported pain intensity scores 0-10 point with a Numerical Rating Scale (NRS)<br>• Participants were requested to report their pain scores 3 times a day at least 4 hours apart<br>• Participants could submit additional reports as well<br>• Participants also received automatic electronic reminders 3 times a day to log pain score<br>• All reports were electronically time stamped and recorded on a secure database<br>• e-diary allowed participants to see their previously reported pain scores<br>• Text messaging was allowed to report pain if patients unable to use the web-based system<br>• Hospital pain records were also collected from medical records<br>• Research staff contacted participants when their pain intensity score was higher than their predetermined medical intervention level<br>• Token monetary incentives were planned to encourage adherence to e-Diary
Jacob, 2013 [59]	**e-Diary or web-based intervention**<br>• Patients accessed the platform twice daily<br>• Participants reported:<br>o Symptoms Checklist (total of 27 symptoms)<br>o Visual Analog Scales (VAS) for pain<br>o Adolescent Pediatric Pain Tool for marking the locations and the quality of pain (affective,

	evaluative, sensory, and temporal dimensions of pain)<br>o Medications checklist (name, dose, and frequency of medications taken for pain), and the mount of relief received from medications<br>o Non-pharmacological strategies that were used to relieve pain and the amount of relief received from these strategies<br>o Sleep<br>o Thoughts/Feelings checklist<br>o Healthcare visits<br>• Advanced provider nurse (APN) with expertise in SCD remotely monitored platform<br>• The APN contacted participants (via text messages or phone calls) in the event that pain and other symptoms required attention, to maintain contact and monitor their progress<br>• Smartphones with wireless service plans were provided at no cost
Gallo, 2014 [56];<br>Wilkie, 2013 [65]	**CHOICES intervention**<br>• Wed-based, tailored, multimedia education program on reproductive options/consequences<br>• For men and women with sickle cell disease (SCD) and sickle cell trait (SCT)<br>• The goal is to develop and implement a parenting plan that supports their informed reproductive health decision and reproductive health behaviors<br>• Highly interactive and experiential program<br>• Required an average of 2 hours to complete<br>• Simplified concept of teaching around the circle with four basic concepts:<br>o Concrete experience (video, audio or text-based case study, or clinical practice simulation) where participants were involved in a concrete experience related to reproductive issues relevant to people with SCD or SCT<br>o Reflective observation where participants reflected on the concrete experience from different perspectives (partner, family members, spiritual leader, etc.)<br>o Abstract conceptualization where participants were engage with content about SCD, SCT, and reproductive issues and behaviors<br>o Active experimentation where participant explored the content in video clips of the perspectives of other couples who made a variety of decisions and engaged in a variety of behaviors and discussed the consequences they faced
Hazzard, 2002 [67]	**STARBRIGHT World**<br>• Curriculum:<br>o Health-education information from reliable and accurate websites and programs developed by STARBRIGHT Foundation (e.g. Sickle Cell Slime-O-Rama Game)

	o Functionality of having children and teens being able to interact with other hospitalized children through computer systems, such as video conferencing, chat rooms, and e-mail<br>o Distraction and entertainment during inpatient stay via different recreational activities, such as online games, arts and crafts projects<br>• STARBRIGHT World is a private computer network developed for hospitalized children by the STARBRIGHT Foundation<br>• Two versions of the curriculum for patients age 8-12 and 13-18 years old<br>• 3 educational sessions (45 min for Session 1, and 15 min for Sessions 2 and 3)<br>• Participants completed a worksheet for every day of the curriculum<br>• Research staff received training on the curriculum and different related activities
Yoon, 2007 [66]	**CD-ROM educational game “Sickle Cell Slime-O-Rama Game ®”**<br>• Part of the starlight Series (www.starlight.org)<br>• Seven questions per round, 2-3 choices per question<br>• Questions included 3 categories:<br>o Sickle cell disease facts<br>o Taking care of your health<br>o Pain management<br>• With each answer, the program indicated whether the choice was correct or not<br>The program provided the right answer with an explanation if it was not correct
